# Comparative Proteomic Analysis of Two Differently Extracted Coptis chinensis in the Treatment of Type 2 Diabetic Rats

**DOI:** 10.1155/2018/3248521

**Published:** 2018-09-13

**Authors:** Xin Qiu, Xinyu Wei, Hongwei Guan, Hao Su, Jing Gong, Ke Fang, Xin Zou, Hui Dong, Lijun Xu, Fuer Lu

**Affiliations:** ^1^Institute of Integrated Traditional Chinese and Western Medicine, Tongji Hospital, Tongji Medical College, Huazhong University of Science and Technology, Wuhan 430030, China; ^2^Accounting Department, Lowa State University, Lowa State 50011, USA; ^3^Department of Integrated Traditional Chinese and Western Medicine, Tongji Hospital, Tongji Medical College, Huazhong University of Science and Technology, Wuhan 430030, China

## Abstract

Coptis chinensis (CC) is widely used to treat diabetes in traditional Chinese medicine due to its significant hypoglycemic and hypolipidemic effects. It was reported that CC powders are more effective than CC decoctions. In this study, a rat model of type 2 diabetes was established and treated with supercritical-extracted CC and gastric juice extracted CC, respectively. Body weight, fasting plasma insulin, insulin resistance index, and lipid profiles were measured along with oral glucose tolerance tests (OGTTs). In addition, the levels of plasma proteins were compared between type 2 diabetic rats and CC-treated rats using an iTRAQ-based quantitative proteomic analysis. The results showed that the plasma levels of triglyceride (TC), total cholesterol (TG), and low-density lipoprotein (LDL) in rats of both CC-treated groups were significantly decreased. In addition, the proteomic analysis identified 929 proteins, while 15 proteins were selected from these 929 proteins based on their expression levels and bioinformatic results. Among these 15 proteins, 9 proteins (IGF-1, Igfbp4, Igfbp-6, Igfals, C2, C4, Cfi, Prdx-2, and Prdx-3) were upregulated in the two CC-treated groups, while 6 proteins (Pla2g7, Pcyox1, ApoC-1, ApoC-3, ApoB-100, and ApoE) were downregulated. The functions of these proteins are associated with glucose metabolism, insulin action, immunity, inflammation, lipid metabolism, oxidation, and antioxidation. The two differently extracted CC did not show significant differences in terms of their treatment efficacy. This research expanded our understanding on the therapeutic effects and mechanisms of CC in the treatment of type 2 diabetes.

## 1. Introduction

The number of diabetic patients in the past 35 years has increased by four times. It was estimated that the global incidence of diabetes was about 9% in 2014 [1]. In China, the incidence of adult diabetes reached 11.6% and the rate of prediabetes was 50.1%. However, only 25.8% of diabetics received hypoglycemic treatment, and only 39.7% of treated diabetics achieved successful glucose control [2]. It has been reported that poor control of blood glucose led to the deaths of 370 million diabetics in 2012 [3]. High mortality of diabetes has become a global health problem and puts a huge economic burden on the world population, urging researchers to clarify the pathogenesis of diabetes using advanced techniques.

Proteomic technologies are large-scale research tools that can provide abundant data regarding the pattern of protein expression and are widely used to explore the molecular mechanisms underlying the function of complex bioactive mixtures, including traditional Chinese medicine (TCM). Recently, a new method, isobaric tags for relative and absolute quantity (iTRAQ), has been used to simultaneously measure protein content in multiple test samples. As an automatic, multidimensional, and more sensitive tool, iTRAQ labeling coupled with liquid-phase secondary mass spectrometry (LC-MS/MS) is more suitable for the study of pathogenic mechanisms and pathophysiology of diseases [4, 5].

**Figure 1:**
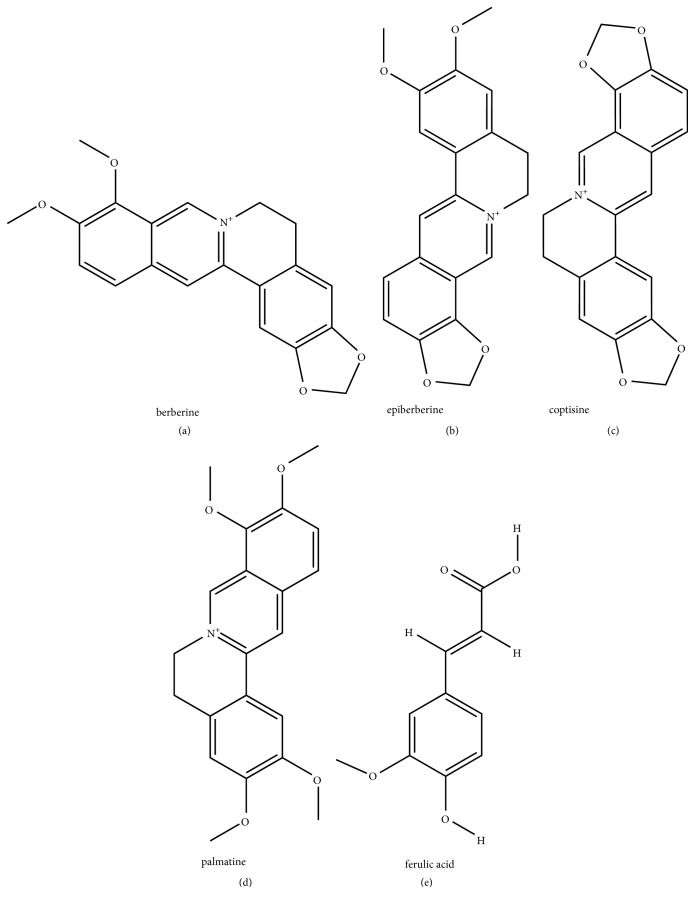
**The main component of Coptis chinensis: (a)** berberine;** (b) **epiberberine;** (c) **coptisine;** (d) **palmatine;** (e) **ferulic acid.

Coptis chinensis (CC, Huanglian in Chinese) has been used to treat diabetes for thousands of years in China. The main components of CC are berberine, epiberberine, coptisine, palmatine, ferulic acid, and berberrubine (Figure 1) [6]. Berberine has been proved to reduce insulin resistance and promote insulin secretion [7]; epiberberine, coptisine, and palmatine exert a strong effect on aldose reductase inhibition [8]; jatrorrhizine and ferulic acid [9, 10] also play a certain hypoglycemic and lipid-regulating role. All above components might contribute to the effect of CC in the treatment of type 2 diabetes mellitus (T2DM). In addition, evidence has shown that CC powders have better pharmacokinetic properties than CC decoctions. For example, our previous work showed that, as compared with a CC decoction, CC powders demonstrated higher bioavailability, slower in vivo elimination, and better absorption and distribution profiles [11]. CC powders also exert excellent hypoglycemic and lipid-lowering effects [12]. However, the mechanisms underlying the functions of CC and its molecular targets in the treatment of T2DM remain unclear.

In this study, the iTRAQ technology was used to compare the effect of CC obtained by two different extracted methods in the treatment of diabetic rats. In addition, differentially expressed proteins in the plasma of diabetic rats and CC-treated rats were identified using a proteomic technology. At the same time, the potential pathways of these proteins implicated in the pathogenesis of T2DM were elucidated using pathway and network analyses.

## 2. Materials and Method

### 2.1. Substances

Dried rhizoma of CC was purchased from Hubei Herbal Medicine Company (Wuhan, China) and was identified by Prof. Zhang Xiu-Qiao from Hubei University of Chinese Medicine as Coptis chinensis Franch, which was called “Wei Lian” in Chinese. The purchased CC was ground into powders and kept in a desiccator at ambient temperature. In order to obtain homogenous powders, the dried powders were filtered through a sieve of 450 ± 20 *μ*m meshes.

Supercritical extraction of CC was carried out as follows: 200 g of CC powder was placed into an extraction kettle (HA221-40-11-C supercritical fluid extractor, Nantong, China). The extraction conditions were as follows: extraction time, 5 min of static extraction followed by up to 2 h of dynamic extraction; temperature, 50°C; pressure, 200 Mpa; entrainer, 300 ml of 90% methanol; flow rate of carbon dioxide (gaseous state), 30 L/h; temperature or extraction and separation: 45°C. With the 200 g input, about 11.59 g of dried powder was extracted and stored in a refrigerator at 4°C before use.

Gastric juice extracted CC was prepared as follows: 20 g of CC powder was mixed with an artificial gastric juice (pH 1.2, obtained by dissolving 2.0 g of sodium chloride in 7.0 mL of hydrochloric acid and further diluted with water to a final volume of 1,000 mL [13]) for 30 min at 37°C. Subsequently, the mixture was filtered twice and the filtrate was combined. After being evaporated in a rotary evaporator and freeze-dried, about 4.00 g of extract were obtained and stored at 4°C for subsequent use.

### 2.2. Animals, Groups, and Intervention

Male Wistar rats with a weight of between 190 and 210 g were purchased from WeiTongLiHua Co., Ltd, and housed in SPF Grade facilities in the experimental animal center of Huazhong University of Science and Technology (SCXK (Jing)2012-0001). After 10 days of acclimatization, the rats were randomly divided into a normal group (N group) and a high glucose fatty diet group (HGFD group). The rats in the N group was fed with a standard diet (containing 35% flour, 20% corn meal, 20% soy meal, 15.5% bran, 5% fish meal, 1% dusty yeast, 0.5% bean oil, 2.5% bone meal, and 0.5% salt), while the diet in the HGFD group contained 67.5% of the standard diet, 12% lard, 2% cholesterol, 0.5% bile salts, and 20% sugar. The dietary regimens of the rats in different groups were maintained the same throughout the entire experiment. Forty days later, each rat in the HGFD group received an intravenous injection of 24 mg/kg streptozotocin (STZ) [14]. Subsequently, the normal 95% confidence intervals (CIs) at various time points of the oral glucose tolerance test (OGTT) were calculated based on the blood glucose level of the rats in the N group. If the blood glucose was greater than 20% of the upper limit of CI at any time point, the rats were considered as successfully modeled diabetic rats. These diabetic rats were then randomly divided into a model group (M group) and different treatment groups, with 15 rats in each group. All experiments were approved by the animal ethics committee of Huazhong University of Science and Technology (No. 2016S615).

The treatment groups included a group treated by CC obtained using supercritical extraction (A group), a group treated by CC obtained using artificial gastric juice extraction (B group), and a group treated by metformin (D group). All test compounds were dissolved in a 1: 1 oil-water mixture. CC obtained using supercritical extraction was administered in the A group at a dose of 85mg/kg/day. CC obtained using artificial gastric juice extraction was administered in the B group at a dose of 333 mg/kg/day [15]. In the D group, the daily dose of metformin was 184 mg/kg. Daily lavage volume of each rat was 1 ml/100 g body weight, and the solution concentration was calculated according to the lavage volume. In addition, a 1:1 mixture of oil and water was given to the rats in N and M groups via intragastric administration. The doses of gastric irrigation were given for 9 weeks and adjusted weekly based on the weight of the rats.

### 2.3. OGTT, Plasma Lipid, and Insulin Measurement

After the interventions were carried out, all rats were subjected to OGTT. During OGTT, the rats were fasted for 12 h before 2 g/kg of glucose was administrated. Tail vein blood samples were collected from each rat at 0, 60, and 120 min after the intragastric administration to detect the blood glucose levels using a glucose monitor (Roche, German).

After the experiment was completed, all animals were anesthetized by intraperitoneal injection of 50 mg/kg pentobarbital sodium. Abdominal aorta blood samples were then collected using heparin sodium anticoagulant tubes. Subsequently, the blood samples were centrifuged at 3000 rpm for 10 min to separate plasma. A part of the separated plasma was used to measure the levels of lipids, while the remaining part of the separated plasma was rapidly frozen under liquid nitrogen and stored at −80°C for proteomic analysis.

The concentration of plasma insulin was measured using a Rat/Mouse Insulin ELISA kit (Millipore, German). For the detection of triglyceride (TG) content and total cholesterol (T-CHO), a colorimetric assay (Jiancheng, Nanjing, China) was used. A double reagent direct method (Jiancheng, Nanjing, China) was used to detect the level of low-density lipoprotein (LDL).

### 2.4. Protein Extraction, Digestion, and iTRAQ Labeling

The plasma samples were transferred into 5-mL centrifuge tubes, which were then centrifuged at 12,000 g and 4°C for 10 min to collect the supernatant. Subsequently, the samples were processed by a ProteoMiner™ Protein Enrichment Small-Capacity Kit (Bio-Rad, US) to enrich low abundance proteins. Finally, the protein concentration was determined with a BCA kit (Biyuntian, China) according to the manufacturer's instructions.

For digestion, the protein solution was reduced with 5 mM dithiothreitol for 30 min at 56°C and alkylated with 11 mM iodoacetamide for 15 min at room temperature away from light. Each protein sample was then diluted by 100 mM TEAB until the urea concentration was less than 2M. Finally, trypsin was added to the samples at a 1:50 trypsin-to-protein mass ratio and incubated overnight to carry out the first digestion, followed by another 4 h digestion using a 1:100 trypsin-to-protein mass ratio.

After trypsin digestion, peptides were desalted by a Strata X C18 SPE column (Phenomenex), vacuum-dried, reconstituted in 0.5 M TEAB, and processed with an iTRAQ kit according to the manufacturer's protocol. In brief, one unit of iTRAQ reagent was thawed and reconstituted in acetonitrile. The peptide mixtures were then incubated with the iTRAQ reagent for 2 h at room temperature, pooled, desalted, and dried by vacuum centrifugation.

### 2.5. HPLC Fractionation

The tryptic peptides were fractionated into fractions by high pH reverse-phase HPLC using an Agilent 300Extend C18 column (5 *μ*m particles, 4.6 mm ID, 250 mm length). In brief, peptides were initially separated into 60 fractions over 60 min with 8% to 32% gradient acetonitrile (pH 9.0). Subsequently, the peptides were combined into 18 fractions and dried by vacuum centrifugation.

### 2.6. LC-MS/MS Analysis

The tryptic peptides were dissolved in 0.1% formic acid (solvent A) and then directly loaded onto a homemade reversed-phase analytical column (15-cm length, 75 *μ*m ID). The gradient elution included an increase from 6% to 23% of solvent B (0.1% formic acid in 98% acetonitrile) over 26 min, followed by an increase from 23% to 35% of solvent B in 8 min, an increase to 80% of solvent B in 3 min, and holding at 80% of solvent B for 3 min. All elution was carried out at a constant flow rate of 400 nL/min on an EASY-nLC 1000 UPLC system.

The peptides were ionized by an NSI source and analyzed online by Q ExactiveTM Plus (Thermo) tandem mass spectrometry (MS/MS) coupled to UPLC. The electrospray voltage was 2.0 kV. The m/z scan range was 350 to 1800 for full scan, and intact peptides were detected in the Orbitrap at a resolution of 70,000. Peptides were subsequently selected for MS/MS using an NCE setting of 28, and the fragments were detected in the Orbitrap at a resolution of 17,500. A data-dependent procedure was used and contained cycles of one MS scan followed by 20 MS/MS scans with 15.0 s dynamic exclusion. Automatic gain control (AGC) was set at 5E4. Fixed first mass was set as 100 m/z.

### 2.7. Database Search

The resulting MS/MS data were processed using a Maxquant search engine (v.1.5.2.8). Tandem mass spectra were searched against the UniprotKB Rattus norvegicus database, which covered 35842 of protein sequences concatenated with reverse decoy database. Trypsin/P was specified as the cleavage enzyme and up to 2 missing cleavages were allowed. The range of the initial search was set to 5 ppm for precursor ions, while the range of the primary search was set to 5 ppm and 0.02 Da for fragment ions. Carbamidomethyl on Cys was specified as fixed modification, while the oxidation on Met was specified as variable modifications. FDR was adjusted to < 1% and minimum score for peptides was set to > 40. To evaluate the significant changes in protein expression, cut-off values of > 1.5- or < 0.667-fold changes in protein expression were used.

### 2.8. Bioinformatics Methods

#### 2.8.1. Annotation Methods

*GO Annotation. *Gene Ontology (GO) annotation proteome was derived from the UniProt-GOA database (www.http://www.ebi.ac.uk/GOA/). Firstly, each identified protein ID was converted to a UniProt ID and subsequently mapped to a GO ID based on the protein ID. If some identified proteins were not annotated by the UniProt-GOA database, InterProScan software would be used to annotate the GO functions of the proteins using a protein sequence alignment method. Subsequently, the proteins were classified by Gene Ontology annotations based on three categories: biological process, cellular component, and molecular function.

*Domain Annotation. *Using the InterPro domain database (http://www.ebi.ac.uk/interpro/), a freely accessible database that integrates diverse information about protein families, domains, and functional sites, the domain functional descriptions of identified proteins were annotated by InterProScan (a sequence analysis tool) based on a protein sequence alignment method. The key elements of the InterPro domain database are diagnostic models, known as signatures, against which protein sequences can be mapped to determine their potential functions. InterPro has been used in the large-scale analysis of whole genomes and metagenomes, as well as in characterizing individual protein sequences.

*KEGG Pathway Annotation.* Kyoto Encyclopedia of Genes and Genomes (KEGG) database was used to annotate protein pathways. Firstly, using KAAS, a KEGG online service tool, the KEGG database description of the proteins was annotated. Subsequently, the annotation results were mapped to the KEGG pathway database using KEGG mapper, a KEGG online service tool.

#### 2.8.2. Functional Enrichment

*Enrichment of Gene Ontology. *Proteins were classified by GO annotation into three categories: biological process, cellular compartment, and molecular function. For each category, a two-tailed Fisher's exact test was employed to test the enrichment of the differentially expressed protein against all identified proteins. The GO with a corrected p value of < 0.05 was considered significant.

*Enrichment of Pathways*. Encyclopedia of Genes and Genomes (KEGG) database was used to identify enriched pathways by a two-tailed Fisher's exact test, so that the enrichment of differentially expressed proteins could be tested against all identified proteins. The pathways with a corrected p value of < 0.05 were considered significant. These pathways were then classified into hierarchical categories according to the instruction on the KEGG website.

*Enrichment of Protein Domains. *For the proteins in each category, the InterPro (a tool used for the functional analysis of protein sequences by classifying them into families and by predicting the presence of domains and important sites) database was searched and a two-tailed Fisher's exact test was employed to test the enrichment of differentially expressed proteins against all identified proteins. Protein domains with a p value of < 0.05 were considered significant.

#### 2.8.3. Enrichment-Based Clustering

For further hierarchical clustering based on different functional classifications of proteins (such as GO, Domain, Pathway, and Complex), all categories obtained after enrichment were first collated along with their P values and subsequently filtered to select categories which were at least enriched in one of the clusters with a P value of <0.05. This filtered matrix of P values was then transformed using the formula x = −log⁡10 (P value). Finally, these x values were z-transformed for each functional category and the z scores were subsequently clustered by one-way hierarchical clustering (Euclidean distance, average linkage clustering) in Genesis. Cluster memberships were visualized by a heat map using the “heatmap.2” function in the “gplots” R-package.

### 2.9. Statistical Analysis

Statistical analyses were performed with GraphPad Prism version 5.01 (GraphPad Software, Inc., San Diego, CA, USA). The variables in each group were tested to determine if they were normally distributed. Analysis of variance was used for the comparisons of sample means among multiple groups. The SNK method was used for pairwise comparison. *P* < 0.05 was considered to be significant.

## 3. Results

### 3.1. CC Decreases Plasma Glucose and Lipid in Diabetic Rats

**Figure 2:**
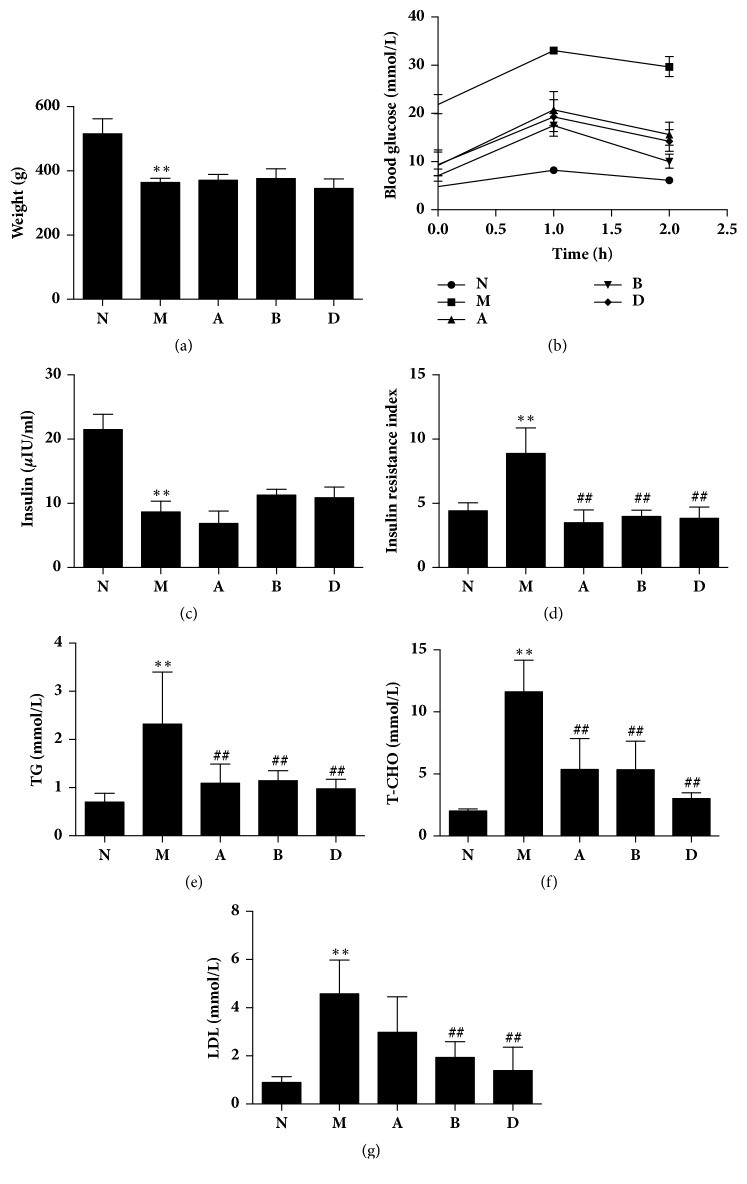
**Coptis chinensis (CC) is effective in decreasing plasma glucose and lipid. (a)** Body weight of rats in each group;** (b) **OGTT of rats after oral gavage with 2.0 g / kg glucose. Data is expressed as mean ± SE. P <0.01 for each of the N versus M, A versus M, B versus M, and D versus M group at each time point;** (c) **plasma insulin concentration in the rat;** (d) **IRI after treatment;** (e) **plasma TG concentration;** (f) **plasma T-CHO concentration;** (g) **plasma LDL concentration. N: normal control group; M: diabetic model group; A: supercritical extraction of Coptis chinensis group; B: simulated gastric juice extraction of Coptis chinensis group; D: metformin treatment group; mean ± SD of data except OGTT. n=6 rats in each group. ^*∗∗*^P <0.01 relative to group N, ^##^P<0.01 relative to group M.

Consistent with a previous study, diabetic rats showed weight loss, abnormal OGTT results, an elevated plasma lipid level, and a reduced concentration of fasting plasma insulin [16]. CC intervention did not significantly increase body weight and fasting concentration of plasma insulin. However, CC reversed elevated levels of fasting and postprandial blood glucose, plasma TG, and T-CHO in diabetic rats. Furthermore, CC decreased insulin resistance index (IRI) as well as LDL in diabetic rats. Among the above indicators, there were no significant differences between the two extracted CC. These data indicated that CC exerted beneficial effects on metabolic parameters (Figure 2).

### 3.2. Comparative Analysis of Plasma Proteomic Changes in Each Group

This study measured a total of 929 proteins, of which 789 proteins produced quantitative information. In this study, more than 1.5 times increase in protein expression was considered as a significant increase, while a reduction in protein expression to a level less than 0.67 of the normal level was considered as a significant reduction. Subsequently, in the N and M groups, the expression of 115 proteins was upregulated and the expression of 116 proteins was downregulated. In the A and M groups, the expression of 101 proteins was upregulated and the expression of 59 proteins was downregulated. In both N and M groups, the expression of 70 proteins was upregulated and the expression of 58 proteins was downregulated. In both B and M groups, the expression of 109 proteins was upregulated and the expression of 71 proteins was downregulated (Table 1).

### 3.3. Functional Classification and Functional Enrichment of GO Annotation

Based on the above data, a bioinformatics analysis of proteins containing quantitative information, including protein annotation, functional classification, and functional enrichment, was carried out systematically.

#### 3.3.1. N versus M Group

**Figure 3:**
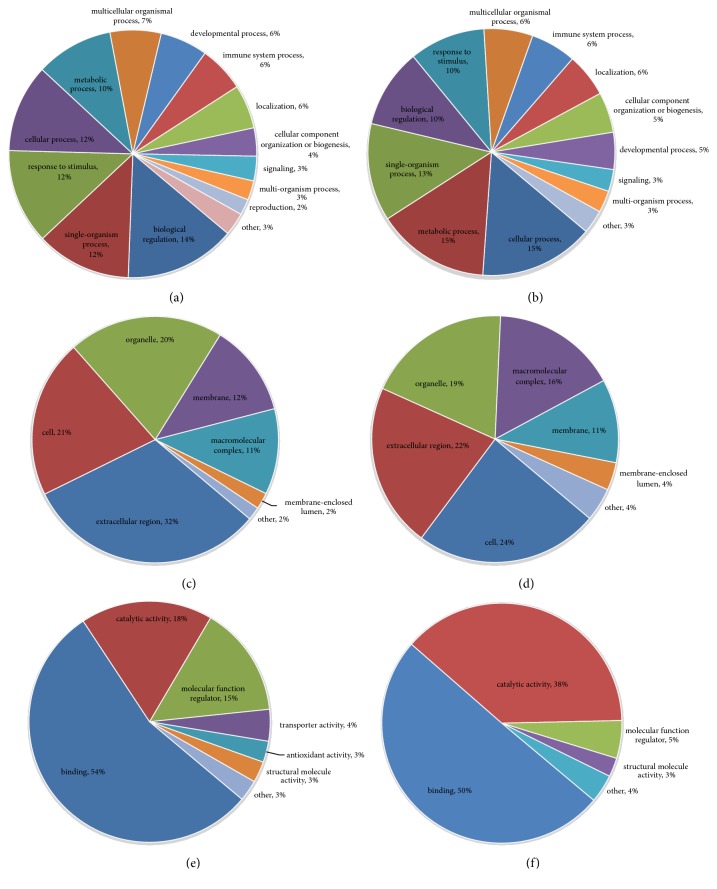
**Normal group versus model group (N versus M) Go analysis information. (a)** Biological process: upregulated proteins: the response to biological regulation (14%) was the major component.** (b) **Biological process: downregulated proteins: the responses to cellular processes (15%) and metabolic processes (15%) were the dominant features.** (c) **Cellular components: upregulated proteins: extracellular region (32%) formed the main component of the cellular component category.** (d) **Cellular components: downregulated proteins: cell (24%) was the major component.** (e) **Molecular function: upregulated proteins: binding (54%) was the dominant molecular function in the GO assignments.** (f) **Molecular function: downregulated proteins: binding represented 50% of the molecular function.

**Figure 4:**
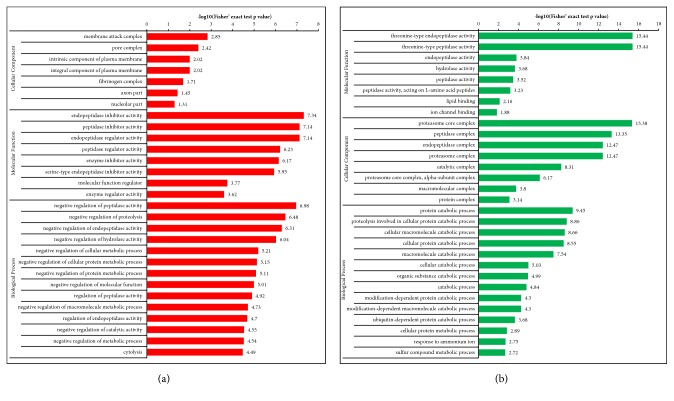
**Enrichment analysis of the GO function annotation of normal versus model group (N versus M). (a) **Upregulated proteins: membrane attack complex, endopeptidase inhibitor activity, and negative regulation of peptidase activity of, respectively, cellular component, molecular function, and biological process were the most representative functions. **(b)** Downregulated proteins: proteasome core complex, threonine-type endopeptidase activity, and protein catabolic process were the most representative functions.

Differentially expressed proteins in the N and M groups were categorized based on the results of GO enrichment analysis. In terms of biological processes, 14% of the proteins with elevated expression were related to each of the “biological regulation”, “single-organism process”, “response to stimulus”, and “cellular process” (Figure 3(a)), while each of “cellular process” and “metabolic process” involved 15% of the proteins with decreased expression. Around 13% of the proteins with decreased expression were related to “single-organism process” (Figure 3(b)). In terms of cellular components, 32% and 21% of the proteins with elevated expression were related to the extracellular region and cells, respectively, followed by the proteins involving organelle (20%; Figure 3(c)). Approximately 24% and 22% of the proteins with decreased expression were linked to cell and extracellular region, respectively, followed by the proteins involving organelle (19%; Figure 3(d)). For molecular functions, 54% of the upregulated proteins were associated with binding, followed by the proteins involving catalytic activity (18%; Figure 3(e)). Approximately 50% of the downregulated proteins were associated with binding, while 38% of these proteins were related to catalytic activity (Figure 3(f)). Subsequently, an enrichment analysis of the GO function annotation was performed, and “membrane attack complex”, “endopeptidase inhibitor activity”, and “negative regulation of peptidase activity” were the most common functions of “cellular component”, “molecular function”, and “biological process” of upregulated proteins (Figure 4(a)). For the downregulated proteins, “proteasome core complex”, “threonine-type endopeptidase activity”, and “protein catabolic process” were the most common functions (Figure 4(b)).

#### 3.3.2. A versus M Groups

**Figure 5:**
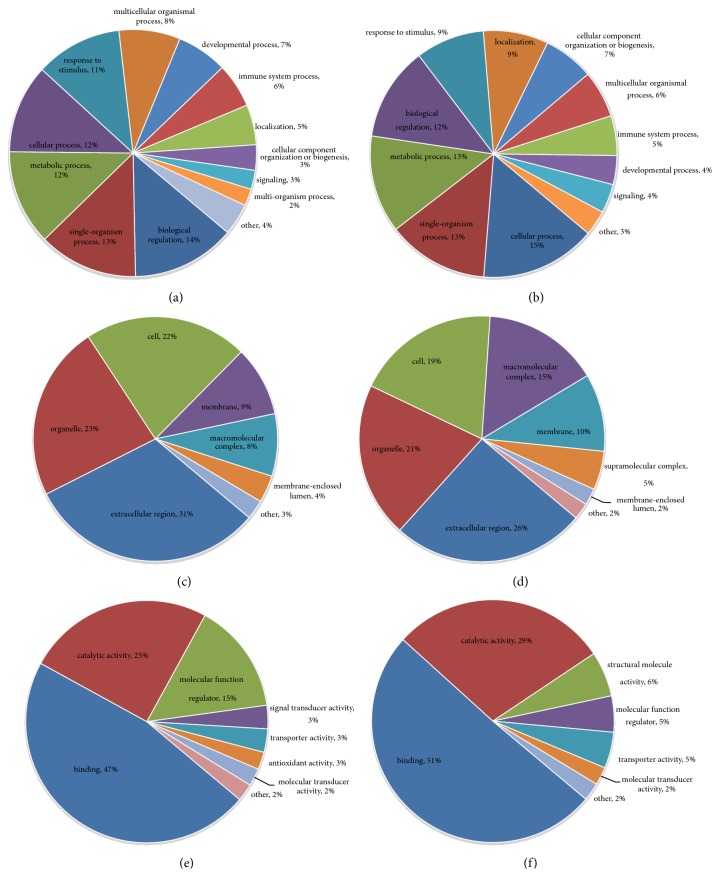
**Supercritical extraction of Coptis chinensis treatment group versus model group (A versus M) Go analysis information. (a) **Biological process: upregulated proteins: the response to biological regulation (14%) was the major component.** (b)** Biological process: downregulated proteins: the response to cellular processes (15%) was the dominant feature.** (c) **Cellular components: upregulated proteins: extracellular region (31%) formed the main component of the cellular component category.** (d) **Cellular components: downregulated proteins: extracellular region (26%) was the major component.** (e) **Molecular function: upregulated proteins: binding (47%) was the dominant molecular function in the GO assignments.** (f) **Molecular function: downregulated proteins: binding represented 51% of the molecular function.

**Figure 6:**
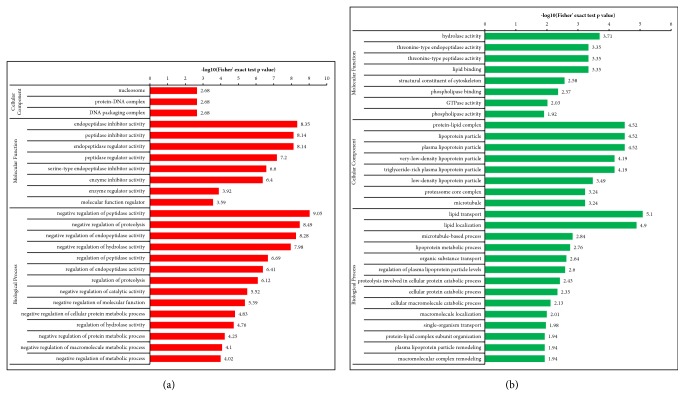
**Enrichment analysis of the GO function annotation of supercritical extraction of Coptis chinensis treatment group versus model group (A versus M). (a)** Upregulated proteins: membrane attack complex, endopeptidase inhibitor activity, and negative regulation of peptidase activity of, respectively, cellular component, molecular function, and biological process were the most representative functions.** (b) **Downregulated proteins: proteasome core complex, threonine-type endopeptidase activity, and protein catabolic process were the most representative functions.

Differentially expressed proteins in the A and M groups were categorized based on the results of GO enrichment analysis. In terms of biological processes, 14% of upregulated proteins were related to “biological regulation”, while 13% were related to each of the “single-organism process”, “metabolic process”, and “cellular process” (Figure 5(a)). Around 15% of the downregulated proteins were related to each of “cellular process”, “single-organism process”, and “metabolic process” (Figure 5(b)). In terms of cellular components, 31% and 23% of upregulated proteins were related to the extracellular region and the organelle, respectively, followed by proteins involving cells (22%; Figure 5(c)). Approximately 26% and 21% of the downregulated proteins were linked to extracellular region and organelle, respectively, followed by proteins involving cells (19%; Figure 5(d)). For molecular functions, 47% of the upregulated proteins were associated with binding, followed by proteins involving catalytic activity (25%; Figure 5(e)). Approximately 51% of downregulated proteins were associated with binding, followed by 29% of downregulated proteins related to catalytic activity (Figure 5(f)). Subsequently, the enrichment analysis of GO function annotation was performed, and “membrane attack complex”, “endopeptidase inhibitor activity”, and “negative regulation of peptidase activity” were the most common functions of “cellular component”, “molecular function”, and “biological process” of upregulated proteins (Figure 6(a)). For downregulated proteins, “proteasome core complex”, “threonine-type endopeptidase activity”, and “protein catabolic process” were the most common functions (Figure 6(b)).

#### 3.3.3. B versus M Groups

**Figure 7:**
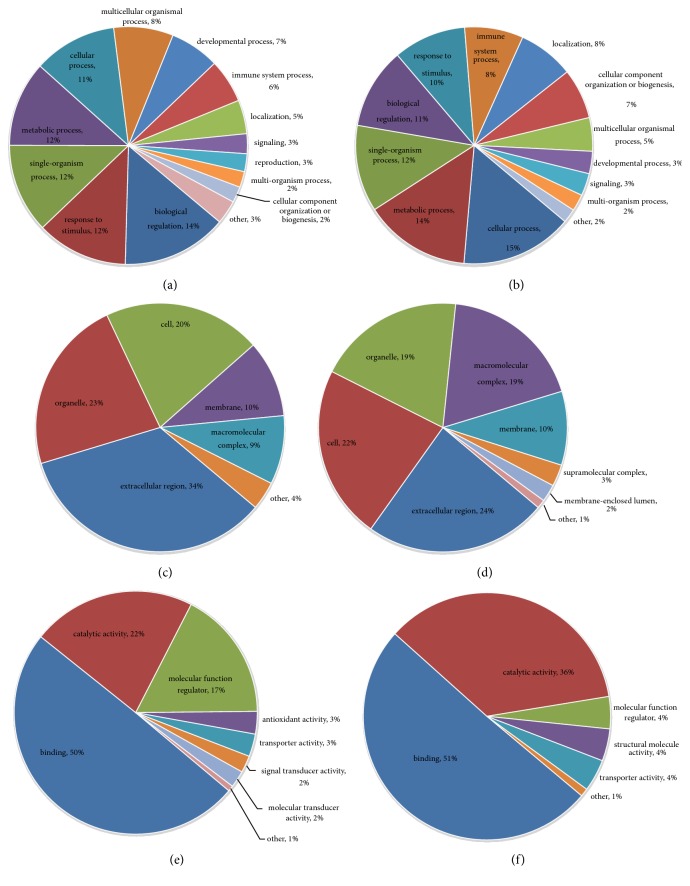
**Artificial gastric juice extraction of Coptis chinensis treatment group versus model group (B versus M) Go analysis information. (a) **Biological process: upregulated proteins: the response to biological regulation (14%) was the major component.** (b) **Biological process: downregulated proteins: the response to cellular processes (15%) was the dominant feature.** (c) **Cellular components: upregulated proteins: extracellular region (34%) formed the main component of the cellular component category.** (d) **Cellular components: downregulated proteins: extracellular region (24%) was the major component.** (e) **Molecular function: upregulated proteins: binding (50%) was the dominant molecular function in the GO assignments.** (f) **Molecular function: downregulated proteins: binding represented 51% of the molecular function.

**Figure 8:**
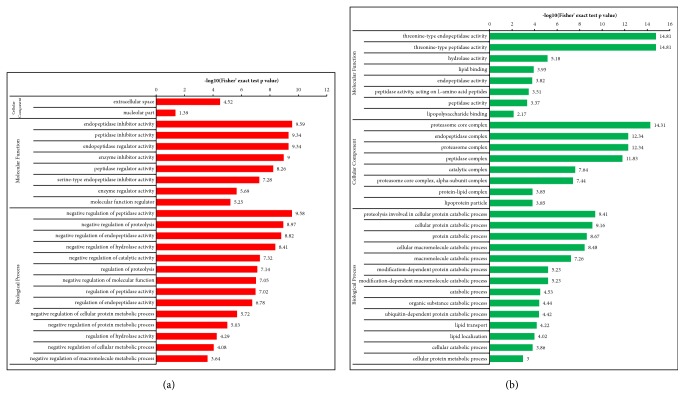
**Enrichment analysis of the GO function annotation of artificial gastric juice extraction of Coptis chinensis treatment group versus model group (B versus M). (a)** Upregulated proteins: extracellular space, endopeptidase inhibitor activity, and negative regulation of peptidase activity of, respectively, cellular component, molecular function, and biological process were the most representative functions.** (b) **Downregulated proteins: proteasome core complex, threonine-type endopeptidase activity, and proteolysis involved in cellular protein catabolic process were the most representative functions.

Differentially expressed proteins in the B and M groups were categorized based on the results of GO enrichment analysis. In terms of biological processes, 14% of upregulated proteins were related to each of “biological regulation”, “single-organism process”, “metabolic process”, and “response to stimulus” (Figure 7(a)). Around 15% of the downregulated proteins were related to each of “cellular process”, “metabolic process”, and “single-organism process” (Figure 7(b)). In terms of cellular components, 34% and 23% of upregulated proteins were related to the extracellular region and the organelle, respectively, followed by proteins involving cells (20%; Figure 7(c)). Approximately 24% and 22% of downregulated proteins were linked to extracellular region and cells, respectively, followed by proteins involving organelle (19%; Figure 7(d)). For molecular functions, 50% of upregulated proteins were associated with binding, followed by proteins involving catalytic activity (22%; Figure 7(e)). Approximately 51% of downregulated proteins were associated with binding, followed by 36% of downregulated proteins related to catalytic activity (Figure 7(f)). Subsequently, an enrichment analysis of GO function annotation was performed, and “extracellular space”, “endopeptidase inhibitor activity”, and “negative regulation of peptidase activity” were the most common functions of “cellular component”, “molecular function”, and “biological process” of upregulated proteins (Figure 8(a)). For downregulated proteins, “proteasome core complex”, “threonine-type endopeptidase activity”, and “proteolysis involved in cellular protein catabolic process” were the most common functions (Figure 8(b)).

#### 3.3.4. A versus B Groups

**Figure 9:**
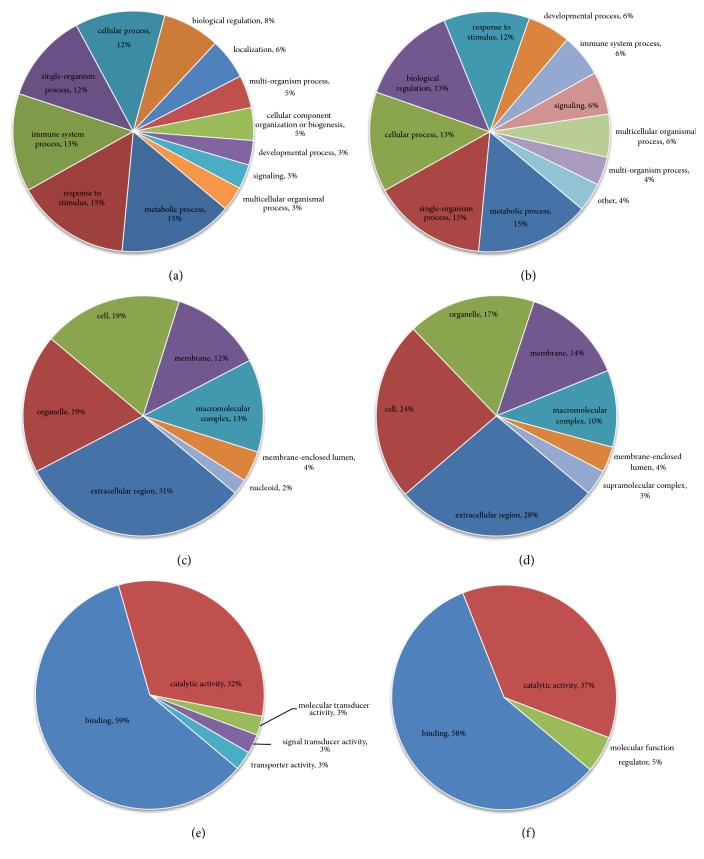
**Supercritical extraction of Coptis chinensis treatment group versus artificial gastric juice extraction of Coptis chinensis treatment group (A versus B) Go analysis information. (a)** Biological process: upregulated proteins: the response to metabolic process (15%) and response to stimulus (15%) were the major component.** (b) **Biological process: downregulated proteins: the responses to metabolic process (15%) and single-organism process (15%) were the dominant features.** (c) **Cellular components: upregulated proteins: extracellular region (31%) formed the main component of the cellular component category.** (d) **Cellular components: downregulated proteins: extracellular region (28%) was the major component.** (e) **Molecular function: upregulated proteins: binding (59%) was the dominant molecular function in the GO assignments.** (f) **Molecular function: downregulated proteins: binding represented 58% of the molecular function.

**Figure 10:**
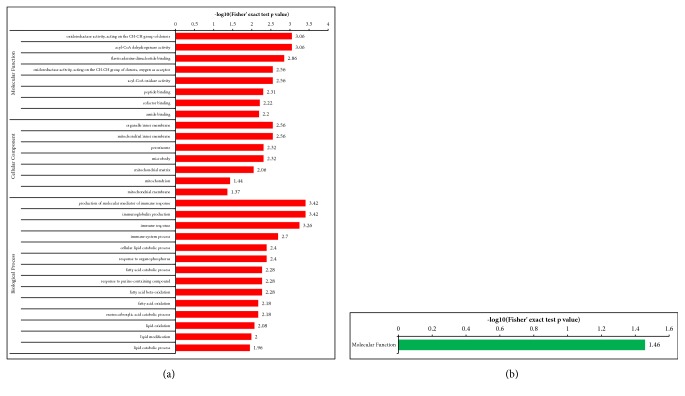
**Enrichment analysis of the GO function annotation of supercritical extraction of Coptis chinensis treatment group versus artificial gastric juice extraction of Coptis chinensis treatment group (A versus B). (a) **Upregulated proteins: oxidoreductase activity, acting on the CH-CH group of donors, organelle inner membrane, and production of molecular mediator of immune response of, respectively, cellular component, molecular function, and biological process were the most representative functions in the upregulated proteins.** (b) **Downregulated proteins: transferase activity was the most representative function in molecular function, and others could not get a valid P value.

Differentially expressed proteins in the A and M groups were categorized based on the results of GO enrichment analysis. In terms of biological processes, 15% of upregulated proteins were related to each of “metabolic process” and “response to stimulus”. 13% of upregulated proteins were related to each of “immune system process”, “single-organism process”, and “cellular process” (Figure 9(a)). Around 15% of the downregulated proteins were related to each of “metabolic process” and “single-organism process”. Around 13% of the downregulated proteins were related to each of “cellular process” and “biological regulation” (Figure 9(b)). In terms of cellular components, 31% and 19% of upregulated proteins were related to the extracellular region and the organelle, respectively, followed by proteins involving cells (19%; Figure 9(c)). Approximately 28% and 24% of downregulated proteins were linked to extracellular region and cells, respectively, followed by proteins involving organelle (17%; Figure 9(d)). For molecular functions, 59% of upregulated proteins were associated with binding, followed by proteins involving catalytic activity (32%; Figure 9(e)). Approximately 58% of downregulated proteins were associated with binding, followed by 37% of downregulated proteins related to catalytic activity (Figure 9(f)). Subsequently, an enrichment analysis of GO function annotation was performed, and “oxidoreductase activity acting on the CH-CH group of donors”, “organelle inner membrane”, and “production of molecular mediator of immune response” were the most common functions of “cellular component”, “molecular function”, and “biological process” of upregulated proteins (Figure 10(a)). For downregulated proteins, “transferase activity” was the most common function of “molecular function”, while other downregulated proteins did not produce a valid P value (Figure 10(b)).

### 3.4. KEGG Pathway Enrichment

**Figure 11:**
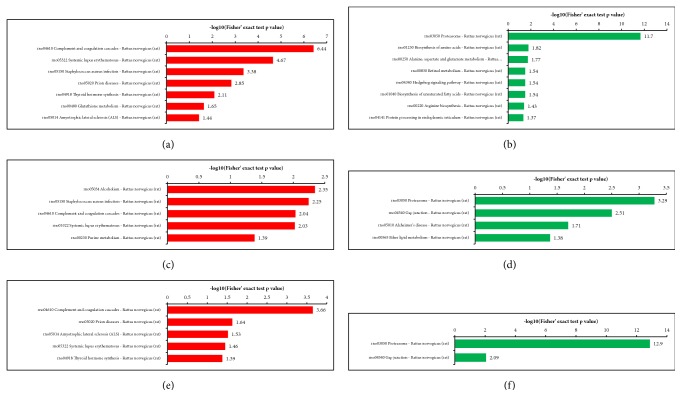
**KEGG Pathway enrichment. (a) **N versus M: KEGG pathway enrichment of upregulated proteins;** (b) **N versus M: KEGG pathway enrichment of downregulated proteins;** (c) **A versus M: KEGG pathway enrichment of increased proteins;** (d) **A versus M: KEGG pathway enrichment of decreased proteins;** (e) **B versus M: KEGG pathway enrichment of upregulated proteins;** (f) **B versus M: KEGG pathway enrichment of downregulated proteins.

In Figure 11, the different protein-enriched KEGG pathways were identified in each group. Differentially expressed proteins in the N and M groups were mapped to multiple pathways in the KEGG database to investigate their biological functions. “Complement and coagulation cascade” was the most common pathway enriched by upregulated proteins, followed by “systemic lupus erythematosus” and “Staphylococcus aureus infection”. The downregulated proteins were significantly enriched in the pathways of “proteasome,” “protein processing in endoplasmic reticulum”, “biosynthesis of amino acids”, and “alanine, aspartate and glutamate metabolism”. Compared with those in A and M groups, the KEGG pathways enriched by upregulated proteins were similar in N and M groups, while the downregulated proteins in N and M groups were significantly enriched in the pathways of “proteasome,” “gap junction,” “Alzheimer's disease,” and “ether lipid metabolism.” Furthermore, in the B and M groups, “complement and coagulation cascade” was the most common pathway enriched by upregulated proteins, followed by “systemic lupus erythematosus” and “Prion diseases”, while the pathways in B and M groups enriched by downregulated proteins were similar with those in A and M groups.

### 3.5. Protein Domain Enrichment

**Figure 12:**
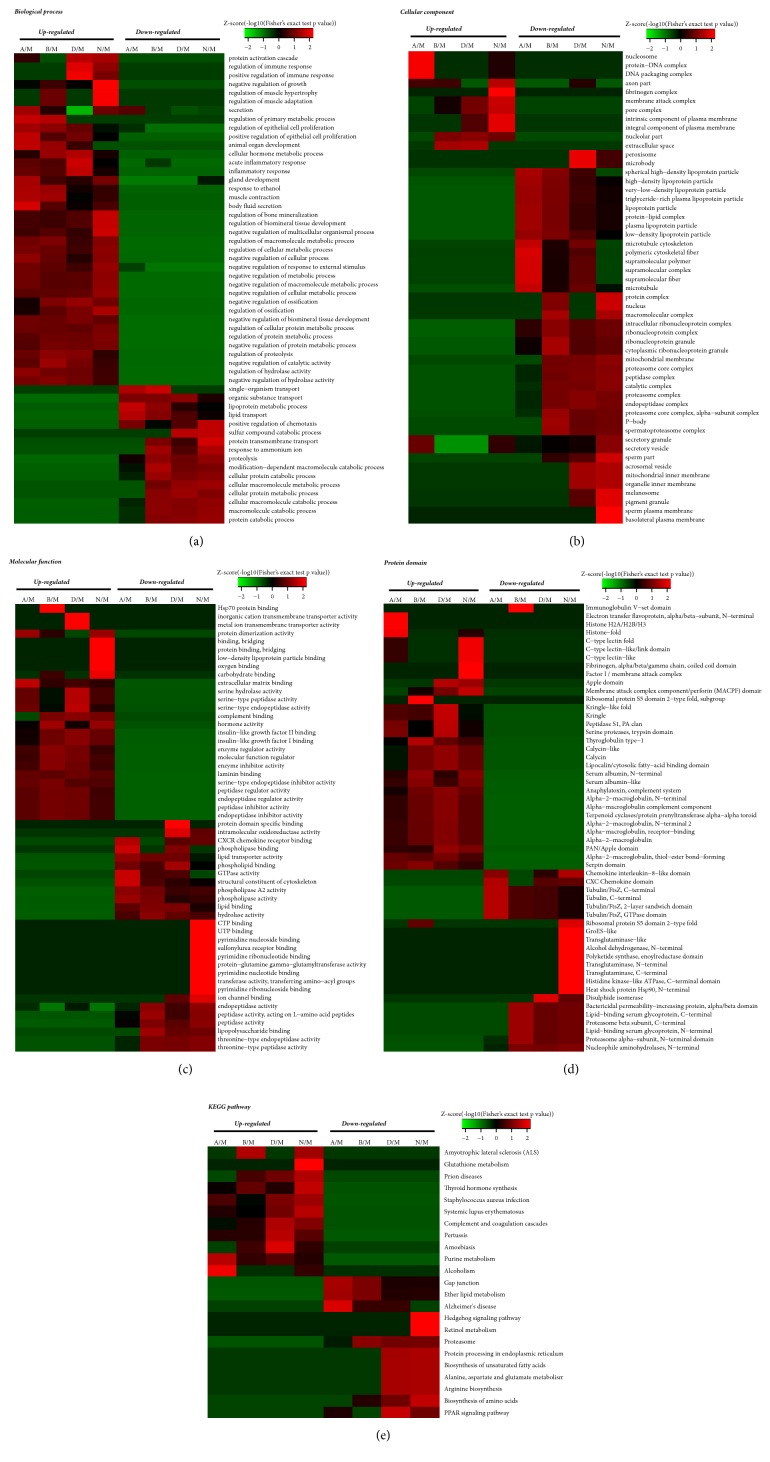
**Heat map obtained from GO and cluster based on feature enrichment. (a)** Biological process analysis;** (b) **cell component analysis;** (c) **molecular function analysis;** (d) **protein domain analysis;** (e) **KEGG pathway analysis. All corrected P values <0.05 passed two-tailed Fisher exact test.

The protein domains refer to some components that repeat in different protein molecules and have similar sequences, structures, and functions. These protein domains are the units of protein evolution. Upregulated proteins in the N group were associated with “Serpin domain,” “Serine proteases, trypsin domain,” and “Peptidase S1, PA clan”, whereas downregulated proteins in the N group were associated with Nucleophile aminohydrolases, N-terminal, Proteasome alpha-subunit, and N-terminal domain. Compared with those in A and M groups, the enriched domains of upregulated proteins in N and M groups were similar, while the downregulated proteins were significantly enriched in domains of “Nucleophile aminohydrolases, N-terminal,” “Tubulin/FtsZ, C-terminal,” “Tubulin/FtsZ, GTPase domain,” “Tubulin/FtsZ, 2-layer sandwich domain,” and “Tubulin, C-terminal.” Upregulated proteins in the B group were associated with “Serpin domain,” “Alpha-2-macroglobulin, N-terminal 2,” “Alpha-macroglobulin, receptor-binding,” and “Alpha-2-macroglobulin”, while the enriched domains of downregulated proteins in the B group were similar to those in A and M groups (Figure 12(d)).

### 3.6. Cluster Analysis Based on Functional Enrichment

The differentially expressed proteins in different groups were classified based on GO annotation, KEGG pathway enrichment, and cluster analysis to determine the correlation in the functions of differentially expressed proteins among different groups (Figure 12).

## 4. Discussion

CC can effectively regulate blood lipid metabolism, improve glucose tolerance, reduce insulin resistance, improve insulin sensitivity, reduce serum inflammatory cytokines, and alleviate lipid peroxidation [7, 17]. In Table 2, the therapeutic targets for CC in the plasma of diabetic individuals, including insulin-like growth factor 1 (IGF-1), insulin-like growth factor binding protein 4 (IGFBP-4), insulin-like growth factor binding protein 6 (IGFBP-6), insulin-like growth factor binding protein, acid labile subunit (IGFALS), complement component 2 (C2), complement component (C4), complement factor I (Cfi), Apolipoprotein C-III (ApoC-III), Apolipoprotein C-I (ApoC-I), Apolipoprotein E (ApoE), Apolipoprotein B-100 (ApoB-100), Peroxiredoxin-2 (PRDX-2), Peroxiredoxin-3 (PRDX-3), prenylcysteine oxidase, and Group VII Phospholipase A2, were identified and validated by proteomic techniques. However, in different CC preparations, there was not much difference in terms of therapeutic targets and efficacy, and the advantages and disadvantages of different CC preparations were not demonstrated in this experiment. To identify therapeutically relevant drug targets of CC in individuals with diabetes, differentially expressed plasma proteins were divided into four regions according to their functions: glucose tolerance and insulin sensitivity, immunity and inflammation, lipoprotein metabolism and transport, and oxidation and antioxidation.

### 4.1. Effects of CC on Glucose Tolerance and Insulin Sensitivity

IGF-1 is an endocrine, paracrine, and autocrine hormone of a 70-amino acid polypeptide that shares structural homology (> 60%) with IGF-2 and proinsulin [18]. IGF-1 plays a number of physiological roles in tissue growth and development, proliferation, lipid metabolism, survival promotion, antiaging, anti-inflammation, anabolic and antioxidant properties, and neurological and hepatic protection [19–24]. IGF-1 exerts a protective effect on mitochondria by protecting it against oxidative damage, increasing ATP synthesis and reducing the production of free radicals in the mitochondria [19–21]. IGF-1 can also promote glucose uptake in some peripheral tissues [25–30]. In addition, exogenous IGF-1 has been shown to lower serum glucose levels [31–33] not only in healthy individuals[34], but also in those with insulin resistance [35–37]. Low circulating levels of IGF-1 have also been associated with reduced insulin sensitivity [38], impaired glucose tolerance, and T2DM[38–40]. The experimental results of this study suggested that the level of IGF-1 in the A and B groups was higher than that in the M group.

In addition, IGF signaling is regulated by six binding proteins, IGFBP1-6. IGF-binding proteins can locally enhance or inhibit the effects of IGF [41]. IGFBP-4 is highly expressed in osteoblasts, while past in vivo studies indicated that IGFBP-4 can potentiate the effects of IGF-1 and IGF-2 [42]. The main function of IGFBP-6 is to inhibit the effects of IGF-II, although IGFBP-6 has no effect on IGF-I [43]. At present, the function of IGFBP-6 is not yet clear, but it may be involved in the pathogenesis of metabolic diseases.

IGFALS encodes a protein acid labile subunit (ALS), which forms a ternary complex with IGF-1, IGFBP-3, and IGFBP-5. The primary role of ALS is to extend the half-life of IGF1 by protecting the ternary complex against degradation [44, 45]. In addition, it was reported that mutations in the IGFALS gene lead to a syndrome of primary IGF-I deficiency [46]. Biallelic mutations in IGFALS may also be associated with insulin resistance [45, 47]. In this study, the expression of IGFBP-4, 6, and IGFALS was significantly increased in the A and B groups. Based on these findings, it is likely that CC can improve glucose tolerance and increase insulin sensitivity by upregulating the expression of IGF-1 and related insulin-like growth factor binding proteins in vivo.

### 4.2. Effects of CC on Immunity and Inflammation

The complement system is a crucial element of the immune defense system. A complex enzyme cascade containing more than 50 circulating and membrane-bound proteins plays an important role in the activation of natural and adaptive immune responses [48, 49]. The complement system is also involved in the process of tissue development, degeneration, and regeneration and plays an important metabolic and inflammatory role in adipose tissues [50]. The clinical status of T2DM is associated with chronic and mild inflammation, such as an increase in serum levels of inflammatory cytokines [51]. In previous proteomics studies, complement-related proteins were upregulated in T2DM [52]. In addition, in some cohort studies, it was found that the levels of complement-related proteins correlate with insulin resistance and elevated blood glucose and hence are considered as risk factors for the development of T2DM [53, 54].

However, in this study, conflicting results were found. In the diabetic model, the complement proteins C2, C4, and Cfi were downregulated. However, after drug intervention (including metformin), these complement proteins were upregulated. In diabetes, the abnormal production or regulation of hundreds of immune mediators leads to changes in metabolic status. Given such complexity, different or even conflicting results are commonly observed [55]. It is believed that the upregulation of complement-associated proteins is most likely reactive. For example, CC may upregulate these proteins to enhance phagocytosis and hypersensitivity without causing direct cell-injury, suggesting that the tissues of the complement system can block the possible role of apoptotic debris and inflammation in tissues [56].

### 4.3. Effects of CC on Lipid Metabolism and Transport

The proteins in plasma lipoproteins are called apolipoproteins and their basic function is to carry lipids and stabilize the structure of lipoproteins. It was found in the present study that the level of apolipoproteins correlates with the level of insulin resistance. Haplotypes in the ApoC-III gene have been shown to lead to elevated ApoC-III levels and an increased susceptibility to type 1 diabetes [57]. ApoC-III gene mutations and ApoC-III levels are also associated with the development of nonalcoholic fatty liver, hepatic insulin resistance, and T2DM [58, 59]. In addition, it has also been reported that ApoC-III, which is produced locally under insulin resistance, is an important agent causing islet *β*-cell dysfunction in diabetes. Reduced ApoC-III in vivo by antisense treatment can improve glucose tolerance [60], and ApoC-I exerts a similar effect as ApoC-III. Prospective studies have shown that ApoC-I levels are higher in polycystic ovarian syndrome (PCOS) patients with insulin resistance [61]. It was also found that severe systemic and hepatic insulin resistance can occur in mice overexpressing ApoC-I [62].

Overproduction of VLDL particles containing hepatic Apo-B-100 has been well documented in animal models and in those with insulin resistance (such as Metabolic Syndrome and T2DM), which can result in the typical dyslipidemia in these disorders [63]. As for ApoE, previous findings suggest that mice carrying the ApoE-3-genotype are more prone to develop impaired glucose tolerance leading to obesity and metabolic complications [64]. In an aging brain, the increasing level of ApoE-4 can further aggravate the inhibitory effect of ApoE-4 on insulin signaling and act as an important pathogenic mechanism underlying insulin resistance [65]. This study showed that the treatment of CC downregulated the expression of above proteins. From these results, it is confirmed that C can reduce the level of lipoproteins, thus improving insulin resistance and glucose tolerance.

### 4.4. Effects of CC on Oxidation and Antioxidation

Oxidative stress is an important pathophysiological mechanism underlying the development and complications of T2DM [66]. A peroxidase system is present in somatic cells and plays a key role in counteracting oxidative stress. PRDX-2 is an antioxidant protein found to be downregulated in patients with T2DM and PCOS. The expression of PRDX-2 indicates the levels of oxidative stress and toxicity in T2DM [67]. Previous data also indicated that PRDX-2 is required for normal insulin secretion [68]. Like PRDX-2, PRDX-3 is also an antioxidant protein. A systemic knockdown of PRDX-3 in mice leads to oxidative stress, increased accumulation of white adipose tissues, dysregulated adiposity, and systemic insulin resistance [69]. In contrast, overexpression of PRDX-3 can reduce the levels of oxidative stress and alleviate insulin resistance, highlighting that oxidative stress plays a key role in maintaining insulin sensitivity [70–74]. In addition, PRDX-3 transcription is downregulated in adipose tissues in obese and insulin-resistant humans, while some studies have shown that diabetics are associated with increased levels of oxidative stress markers such as lipid peroxidation. In addition, PRDX3 can resist oxidative stress [71–77]. The experiment of this study confirmed that CC treatment could increase the expression of PRDX-2 and PRDX-3. It also indicates that CC is likely to improve the oxidation response in diabetes by upregulating the expression of proteins in the peroxidase family.

Prenylcysteine oxidase is detectable both in embryonic tissues and in various adult organs, particularly in the liver. The gene sequence Pcyox1 has also been described as a marker of toxicity in the liver [78]. In addition, Group VII Phospholipase A2 has been described as a novel risk factor for cardiovascular disease [79, 80]. Some reports also showed significant increases in Pla2g7 mRNA expression in the liver of metabolic syndrome rats [81, 82]. In this study, it was found that prenylcysteine oxidase and Group VII Phospholipase A2 were significantly downregulated after CC treatment. It was suggested that CC can improve insulin resistance and treat diabetes by reducing the expression of oxidative factors and increasing the level of antioxidants in the body.

### 4.5. Differences between A and B Groups

No significant difference was found in the two CC treatments in terms of the status of glycemic and lipid disorders. In proteomic analysis, although it was found that 27 proteins were upregulated and 16 proteins were downregulated in group A compared with those in group B, they showed no significant impact on the treatment of T2DM. Therefore, 43 proteins that did not differ between N and M groups were excluded. In addition, the proteins with less than 1.5-fold difference between A versus M and B versus M groups and the proteins with nonmeaningful P values were also eliminated. As a result, only four differentially expressed proteins were found between A and B groups, as shown in Table 3.

Based on the above discussion, IGFBP-4 can enhance the role of IGF-1 and IGF-2 [42], while both types of extracted CC can upregulate IGFBP-4. However, it is worth noting that IGF-1 did not show significant differences in the two CC treatments. Similarly, IGFBP-6 and IGFALS also showed no significant differences in the two CC treatments. Therefore, although the drug in group B can increase the level of IGFBP4, it does not show much difference in the improvement of glucose tolerance and insulin resistance as compared with the drug in group A.

Creatine kinase M-type is a creatine kinase isoenzyme found in muscles. Little information is available regarding the relation of this enzyme to diabetes, but a study using nontargeted proteomics revealed that the expression of creatine kinase M-type in skeletal muscles of women with hyperandrogenism was downregulated, suggesting that the downregulation of this protein may cause insulin resistance [83]. Myosin light chain 1/3 (MLC 1/3) is a member of myosin light chains. A study has shown that muscle fiber components are related to insulin resistance, while exercises can increase the amount of myosin fibers, thereby reducing the risk of diabetes [84]. One study showed that, as compared with the control group, MLC in heart homogenates of diabetic rats was significantly reduced (40% to 45%), and MLC phosphorylation was also significantly reduced (30% to 45%). These results indicate that the decrease of protein content in MLC and the phosphorylation of MLC may lead to diabetic cardiomyopathy [85]. In this study, it was found that both CC A and B can effectively upregulate the expression of creatine kinase M-type and MLC 1/3, which can reduce the risk of diabetes.

As for Protein RGD1311933, it was found in this study that Protein RGD1311933 was upregulated in the M group compared with that in the N group, and the levels of Protein RGD1311933 were reversed after treatment with both types of CC. In addition, B drug decreased the level of Protein RGD1311933 to a greater extent. Although Protein RGD1311933 has yet to have functional annotations in the database, its relationship with diabetes shall be further studied.

In summary, although B drug showed a better efficacy in regulating the levels of above four proteins, the evidence is relatively weak. Therefore, it is very difficult to infer that B drug is more effective than A drug.

## 5. Conclusions

In this experiment, 15 differentially expressed proteins were identified as being related to the efficacy of CC. The functions of these proteins were related to improved glucose, insulin sensitivity, immunity, lipid metabolism, lipid transport, and antioxidant activity. Two differentially extracted CC treatments did not show significant differences in their treatment efficacy. In summary, this study deepens our understanding about the role of CC in the treatment of diabetes.

## Figures and Tables

**Table 1:** Differentially expressed protein summary (P value < 0.05).

**Compared sample name**	**1.5-fold change**	
	**Upregulated**	**Downregulated**
A/M	101	59
B/M	109	71
D/M	93	128
N/M	115	116
A/B	27	16

**Table 2:** Significant differential expressions of proteins in Coptis chinensis-treated rats and the model rats.

**Protein accessio** **n** ^a^	**Protein descriptio** **n** ^b^	**Gene name**	**MW [kDa]**	**A/M Rati** **o** ^c^	**B/M Rati** **o** ^**c**^	**D/M Rati** **o** ^c^	**N/M Rati** **o** ^c^
*Glucose Tolerance and Insulin Sensitivity*							
P35572	Insulin-like growth factor-binding protein 6	Igfbp6	24.193	1.763	2.557	2.181	2.121
A0A0G2JX40	Insulin-like growth factor I	Igf1	16.075	1.422	1.877	1.880	2.001
P21744	Insulin-like growth factor-binding protein 4	Igfbp4	27.745	1.730	2.609	2.527	2.239
F1LRE2	Insulin-like growth factor binding protein, acid labile subunit, isoform CRA_b	Igfals	66.898	1.968	1.973	1.843	2.391

*Immunity and Inflammation*							
P08649	Complement C4	C4	192.16	1.787	1.770	1.868	1.532
Q6MG73	Complement component 2	C2	83.698	1.895	1.748	1.729	1.616
A0A0G2K135	Complement factor I	Cfi	69.774	1.537	1.674	1.808	1.753

*Oxidation and Anti-oxidation*							
A0A0G2JSH9	Peroxiredoxin-2	Prdx2	21.797	1.538	1.723	1.468	2.169
G3V7I0	Peroxiredoxin 3	Prdx3	28.299	2.219	1.992	1.333	2.800
Q5M7T7	Phospholipase A2, group VII (Platelet-activating factor acetylhydrolase, plasma)	Pla2g7	49.49	0.349	0.286	0.234	0.299
Q99ML5	Prenylcysteine oxidase	Pcyox1	56.287	0.530	0.449	0.363	0.371

*Lipid Metabolism and Transport*							
P19939	Apolipoprotein C-I	Apoc1	9.8606	0.498	0.410	0.541	0.581
A0A0G2K8Q1	Apolipoprotein C-III	Apoc3	11.029	0.318	0.284	0.308	0.623
A0A0G2K151	Apolipoprotein E	Apoe	41.199	0.251	0.198	0.203	0.259
F1M6Z1	Apolipoprotein B-100	Apob	509.69	0.426	0.299	0.285	0.286

^a^Database accession numbers; ^b^name and categories of the proteins identified; ^c^ratios of treatments/models.

**Table 3:** Significant differential expressions of proteins in two different extracted CC treatments.

**Protein accessio** **n** ^**a**^	**Protein descriptio** **n** ^**b**^	**Gene name**	**MW [kDa]**	**A/M Rati** **o** ^**c**^	**B/M Rati** **o** ^**c**^	**D/M Rati** **o** ^**c**^	**N/M Rati** **o** ^**c**^	**A/B Rati** **o** ^**c**^
P21744	Insulin-like growth factor-binding protein 4	Igfbp4	27.745	1.730	2.609	2.527	2.239	0.663
A0A0G2JSP8	Creatine kinase M-type	Ckm	43.018	2.655	4.055	2.938	9.693	0.655
P02600	Myosin light chain 1/3, skeletal muscle isoform	Myl1	20.679	1.663	2.610	4.669	2.347	0.637
F1M171	Protein RGD1311933	RGD1311933	38.482	0.451	0.282	0.268	0.254	1.597

^a^Database accession numbers; ^b^name and categories of the proteins identified; ^c^ratios of treatments/models.

## Data Availability

We guarantee the authenticity of the data. If the article is received, we can provide all the original data.

## References

[1] NCD Risk Factor Collaboration (NCD-RisC) (2016). Worldwide trends in diabetes since 1980: a pooled analysis of 751 population-based studies with 4·4 million participants. *The Lancet*.

[2] Xu Y., Wang L., He J. (2013). Prevalence and control of diabetes in Chinese adults. *Journal of the American Medical Association*.

[3] The Lancet (2016). Beat diabetes: an urgent call for global action. *The Lancet*.

[4] Herbrich S. M., Cole R. N., West K. P. (2013). Statistical inference from multiple iTRAQ experiments without using common reference standards. *Journal of Proteome Research*.

[5] Ross P. L., Huang Y. N., Marchese J. N. (2004). Multiplexed protein quantitation in *Saccharomyces cerevisiae* using amine-reactive isobaric tagging reagents. *Molecular & Cellular Proteomics*.

[6] Dai S.-Y., Xu B., Zhang Y. (2016). Establishment and reliability evaluation of the design space for HPLC analysis of six alkaloids in Coptis chinensis (Huanglian) using Bayesian approach. *Chinese Journal of Natural Medicines*.

[7] Leng S. H., Lu F. E., Xu L. J. Therapeutic effects of berberine in impaired glucose tolerance rats and its influence on insulin secretion. *Acta Pharmacologica Sinica*.

[8] Jung H. A., Yoon N. Y., Bae H. J., Min B., Choi J. S. (2008). Inhibitory activities of the alkaloids from Coptidis Rhizoma against aldose reductase. *Archives of Pharmacal Research*.

[9] Ohnishi M., Matuo T., Tsuno T. (2004). Antioxidant activity and hypoglycemic effect of ferulic acid in STZ-induced diabetic mice and KK-Ay mice. *BioFactors*.

[10] Balasubashini M. S., Rukkumani R., Viswanathan P., Menon V. P. (2004). Ferulic acid alleviates lipid peroxidation in diabetic rats. *Phytotherapy Research*.

[11] Wei S. C., Xu L. J., Zou X. Pharmacokinetics of berberine and jateorhizine from Rhizoma coptidis powder and decoction in diabetic rats. *Chinese Hospital Pharmacy*.

[12] Wei S.-C., Xu L.-J., Zou X. (2015). Pharmacokinetic and pharmacodynamic characteristics of berberine and jateorhizine in Coptidis Rhizoma powder and their monomeric compounds in type 2 diabetic rats. *Zhongguo Zhongyao Zazhi*.

[13] JPE C. (2011). *The Japanese Pharmacopoeia*.

[14] Duca F. A., Côté C. D., Rasmussen B. A. (2015). Metformin activates a duodenal Ampk-dependent pathway to lower hepatic glucose production in rats. *Nature Medicine*.

[15] Wei S., Xu L., Zou X. Preliminary study on secreting efects of Rhizoma Coptidis active ingredients and their combina- tions on insulin and glucagon-like peptide1 (GLP-1)in glucose feding mice. *Chinese Hospital Pharmaceutical Journal*.

[16] Wang J., Teng L., Liu Y. (2016). Studies on the antidiabetic and antinephritic activities of paecilomyces hepiali water extract in diet-streptozotocin-induced diabetic sprague dawley rats. *Journal of Diabetes Research*.

[17] Gong J., Hu M., Huang Z. (2017). Berberine Attenuates Intestinal Mucosal Barrier Dysfunction in Type 2 Diabetic Rats. *Frontiers in Pharmacology*.

[18] Rinderknecht E., Humbel R. E. (1978). The amino acid sequence of human insulin-like growth factor I and its structural homology with proinsulin. *The Journal of Biological Chemistry*.

[19] Pérez R., García-Fernández M., Díaz-Sánchez M. (2008). Mitochondrial protection by low doses of insulin-like growth factor-Iin experimental cirrhosis. *World Journal of Gastroenterology*.

[20] Puche J. E., García-Fernández M., Muntané J., Rioja J., González-Barón S., Cortazar I. C. (2008). Low doses of insulin-like growth factor-I induce mitochondrial protection in aging rats. *Endocrinology*.

[21] García-Fernández M., Delgado G., Puche J. E., González-Barón S., Cortázar I. C. (2008). Low doses of insulin-like growth factor I improve insulin resistance, lipid metabolism, and oxidative damage in aging rats. *Endocrinology*.

[22] Conchillo M., de Knegt R. J., Payeras M. (2005). Insulin-like growth factor I (IGF-I) replacement therapy increases albumin concentration in liver cirrhosis: results of a pilot randomized controlled clinical trial. *Journal of Hepatology*.

[23] García-Fernández M., Castilla-Cortázar I., Díaz-Sanchez M. (2005). Antioxidant effects of insulin-like growth factor-I (IGF-I) in rats with advanced liver cirrhosis. *BMC Gastroenterology*.

[24] Castilla-Cortázar I., Pascual M., Urdaneta E. (2004). Jejunal microvilli atrophy and reduced nutrient transport in rats with advanced liver cirrhosis: improvement by Insulin-like Growth Factor I. *BMC Gastroenterology*.

[25] Laager H., Ninnis R., Keller U. (1993). Comparison of the effects of recombinant human insulin-like growth factor-1 and insulin on glucose and leucine kinetics in humans. *The Journal of Clinical Investigation*.

[26] Elahi D., McAloon-Dyke M., Fukagawa N. K. (1993). Effects of recombinant human IGF-I on glucose and leucine kinetics in men. *American Journal of Physiology-Endocrinology and Metabolism*.

[27] Russell-Jones D. L., Bates A. T., Umpleby A. M. (1995). A comparison of the effects of IGF-I and insulin on glucose metabolism, fat metabolism and the cardiovascular system in normal human volunteers. *European Journal of Clinical Investigation*.

[28] Boulware S. D., Tamborlane W. V., Rennert N. J., Gesundheit N., Sherwin R. S. (1994). Comparison of the metabolic effects of recombinant human insulin-like growth factor-I and insulin. Dose-response relationships in healthy young and middle-aged adults. *The Journal of Clinical Investigation*.

[29] Sakai K., Lowman H. B., Clemmons D. R. (2002). Increases in free, unbound insulin-like growth factor I enhance insulin responsiveness in human hepatoma G2 cells in culture. *The Journal of Biological Chemistry*.

[30] Frystyk J., Grøfte T., Skjærbæk C., Ørskov H. (1997). The Effect of Oral Glucose on Serum Free Insulin-Like Growth Factor-I and -II in Healthy Adults. *The Journal of Clinical Endocrinology & Metabolism*.

[31] Zenobi P. D., Guler H.-P., Zapf J., Froesch E. R. (1988). Insulin-like growth factors in the Gottinger miniature-pig. *Acta Endocrinologica*.

[32] Zapf J., Hauri C., Waldvogel M., Froesch E. R. (1986). Acute metabolic effects and half-lives of intravenously administered insulinlike growth factors I and II in normal and hypophysectomized rats. *The Journal of Clinical Investigation*.

[33] Rosenfeld R. G., Hwa V., Wilson E., Plymate S. R., Oh Y. (2000). The insulin-like growth factor-binding protein superfamily. *Growth Hormone & IGF Research*.

[34] Schmid C., Bianda T., Zwimpfer C., Zapf J., Wiesli P. (2005). Changes in insulin sensitivity induced by short-term growth hormone (GH) and insulin-like growth factor I (IGF-I) treatment in GH-deficient adults are not associated with changes in adiponectin levels. *Growth Hormone & IGF Research*.

[35] Zenobi P. D., Glatz Y., Keller A. (1994). Beneficial metabolic effects of insulin-like growth factor I in patients with severe insulin resistant diabetes type A. *European Journal of Endocrinology*.

[36] Saukkonen T., Amin R., Williams R. M. (2004). Dose-dependent effects of recombinant human insulin-like growth factor (IGF)-I/IGF binding protein-3 complex on overnight growth hormone secretion and insulin sensitivity in type 1 diabetes. *The Journal of Clinical Endocrinology & Metabolism*.

[37] Pratipanawatr T., Pratipanawatr W., Rosen C. (2002). Effect of IGF-I on FFA and glucose metabolism in control and type 2 diabetic subjects. *American Journal of Physiology-Endocrinology and Metabolism*.

[38] Succurro E., Andreozzi F., Marini M. A. (2009). Low plasma insulin-like growth factor-1 levels are associated with reduced insulin sensitivity and increased insulin secretion in nondiabetic subjects. *Nutrition, Metabolism & Cardiovascular Diseases*.

[39] Sandhu M. S., Heald A. H., Gibson J. M., Cruickshank J. K., Dunger D. B., Wareham N. J. (2002). Circulating concentrations of insulin-like growth factor-I and development of glucose intolerance: a prospective observational study. *The Lancet*.

[40] Mannino G. C., Greco A., De Lorenzo C. (2013). A fasting insulin-raising allele at IGF1 locus is associated with circulating levels of IGF-1 and insulin sensitivity. *PLoS ONE*.

[41] Tahimic C. G., Wang Y., Bikle D. D. (2013). Anabolic effects of IGF-1 signaling on the skeleton. *Frontiers in Endocrinology*.

[42] Maridas D. E., DeMambro V. E., Le P. T. (2017). IGFBP-4 regulates adult skeletal growth in a sex-specific manner. *Journal of Endocrinology*.

[43] Bach L. A. (2005). IGFBP-6 five years on; not so “forgotten”?. *Growth Hormone & IGF Research*.

[44] Guilin L., Lili N., Haifeng L., Jiazhong G. (2015). Structure and function of insulin-like growth factor acid-labile subunits in mammalian homologues. *Yi chuan*.

[45] Domené H. M., Hwa V., Jasper H. G., Rosenfeld R. G. (2011). Acid-labile subunit (ALS) deficiency. *Best Practice & Research Clinical Endocrinology & Metabolism*.

[46] Poukoulidou T., Kowalczyk J., Metherell L., De Schepper J., Maes M. (2014). A novel homozygous mutation of the IGFALS gene in a female adolescent: Indirect evidence for a contributing role of the circulating IGF-I pool in the pubertal growth spurt. *Hormone Research in Paediatrics*.

[47] Hess O., Khayat M., Hwa V. (2013). A novel mutation in IGFALS, c.380T>C (p.L127P), associated with short stature, delayed puberty, osteopenia and hyperinsulinaemia in two siblings: Insights into the roles of insulin growth factor-1 (IGF1). *Clinical Endocrinology*.

[48] Vlaicu S. I., Tatomir A., Boodhoo D., Vesa S., Mircea P. A., Rus H. (2016). The role of complement system in adipose tissue-related inflammation. *Immunologic Research*.

[49] Lubbers R., van Essen M. F., van Kooten C., Trouw L. A. (2017). Production of complement components by cells of the immune system. *Clinical & Experimental Immunology*.

[50] Phieler J., Garcia-Martin R., Lambris J. D., Chavakis T. (2013). The role of the complement system in metabolic organs and metabolic diseases. *Seminars in Immunology*.

[51] King B. C., Blom A. M. (2017). Non-traditional roles of complement in type 2 diabetes: Metabolism, insulin secretion and homeostasis. *Molecular Immunology*.

[52] Li R., Chen H., Tu K. (2008). Localized-Statistical Quantification of Human Serum Proteome Associated with Type 2 Diabetes. *PLoS ONE*.

[53] Wlazlo N., Van Greevenbroek M. M. J., Ferreira I. (2014). Complement factor 3 is associated with insulin resistance and with incident type 2 diabetes over a 7-year follow-up period: The CODAM study. *Diabetes Care*.

[54] Engström G., Hedblad B., Eriksson K.-F., Janzon L., Lindgärde F. (2005). Complement C3 is a risk factor for the development of diabetes: A population-based cohort study. *Diabetes*.

[55] Hotamisligil G. S. (2017). Foundations of Immunometabolism and Implications for Metabolic Health and Disease. *Immunity*.

[56] Kaye S., Lokki A. I., Hanttu A. (2017). Upregulation of Early and Downregulation of Terminal Pathway Complement Genes in Subcutaneous Adipose Tissue and Adipocytes in Acquired Obesity. *Frontiers in Immunology*.

[57] Hokanson J. E., Kinney G. L., Cheng S., Erlich H. A., Kretowski A., Rewers M. (2006). Susceptibility to type 1 diabetes is associated with apoCIII gene haplotypes. *Diabetes*.

[58] Petersen K. F., Dufour S., Hariri A. (2010). Apolipoprotein C3 gene variants in nonalcoholic fatty liver disease. *The New England Journal of Medicine*.

[59] Perry R. J., Samuel V. T., Petersen K. F., Shulman G. I. (2014). The role of hepatic lipids in hepatic insulin resistance and type 2 diabetes. *Nature*.

[60] Juntti-Berggren L., Berggren P.-O. (2017). Apolipoprotein CIII is a new player in diabetes. *Current Opinion in Lipidology*.

[61] Huang S., Qiao J., Li R., Wang L., Li M. (2010). Can serum apolipoprotein C-I demonstrate metabolic abnormality early in women with polycystic ovary syndrome?. *Fertility and Sterility*.

[62] Muurling M., Van Den Hoek A. M., Mensink R. P. (2004). Overexpression of APOC1 in obob mice leads to hepatic steatosis and severe hepatic insulin resistance. *Journal of Lipid Research*.

[63] Xiao C., Dash S., Morgantini C., Lewis G. F. (2014). New and emerging regulators of intestinal lipoprotein secretion. *Atherosclerosis*.

[64] Slim K. E., Vauzour D., Tejera N., Voshol P. J., Cassidy A., Minihane A. M. (2017). The effect of dietary fish oil on weight gain & insulin sensitivity is dependent on APOE genotype in humanized targeted replacement mice. *The FASEB Journal*.

[65] Zhao N., Liu C.-C., Van Ingelgom A. J. (2017). Apolipoprotein E4 Impairs Neuronal Insulin Signaling by Trapping Insulin Receptor in the Endosomes. *Neuron*.

[66] Evans J. L., Goldfine I. D., Maddux B. A., Grodsky G. M. (2002). Oxidative stress and stress-activated signaling pathways: a unifying hypothesis of type 2 diabetes. *Endocrine Reviews*.

[67] Cortón M., Botella-Carretero J. I., López J. A. (2008). Proteomic analysis of human omental adipose tissue in the polycystic ovary syndrome using two-dimensional difference gel electrophoresis and mass spectrometry. *Human Reproduction*.

[68] Oláhová M., Veal E. A. (2015). A peroxiredoxin, PRDX-2, is required for insulin secretion and insulin/IIS-dependent regulation of stress resistance and longevity. *Aging Cell*.

[69] Huh J. Y., Kim Y., Jeong J. (2012). Peroxiredoxin 3 is a key molecule regulating adipocyte oxidative stress, mitochondrial biogenesis, and adipokine expression. *Antioxidants & Redox Signaling*.

[70] Chen L., Na R., Gu M. (2008). Reduction of mitochondrial H2O2 by overexpressing peroxiredoxin 3 improves glucose tolerance in mice. *Aging Cell*.

[71] Fisher-Wellman K. H., Neufer P. D. (2012). Linking mitochondrial bioenergetics to insulin resistance via redox biology. *Trends in Endocrinology & Metabolism*.

[72] Grimsrud P. A., Picklo M. J., Griffin T. J., Bernlohr D. A. (2007). Carbonylation of adipose proteins in obesity and insulin resistance: Identification of adipocyte fatty acid-binding protein as a cellular target of 4-hydroxynonenal. *Molecular & Cellular Proteomics*.

[73] Monteiro R., Azevedo I. (2010). Chronic inflammation in obesity and the metabolic syndrome. *Mediators of Inflammation*.

[74] Xu H., Barnes G. T., Yang Q. (2003). Chronic inflammation in fat plays a crucial role in the development of obesity-related insulin resistance. *The Journal of Clinical Investigation*.

[75] Jain S. K. (1989). Hyperglycemia can cause membrane lipid peroxidation and osmotic fragility in human red blood cells. *The Journal of Biological Chemistry*.

[76] Peraldi P., Spiegelman B. TNF-alpha and insulin resistance: summary and future prospects. *Molecular and Cellular Biochemistry*.

[77] Hahn W. S., Kuzmicic J., Burrill J. S. (2014). Proinflammatory cytokines differentially regulate adipocyte mitochondrial metabolism, oxidative stress, and dynamics. *American Journal of Physiology-Endocrinology and Metabolism*.

[78] Wouters M. M., Neefs J.-M., Kerchove d'Exaerde A. D., Vanderwinden J.-M., Smans K. A. (2006). Downregulation of two novel genes in Sl/Sld and WLacZ/Wv mouse jejunum. *Biochemical and Biophysical Research Communications*.

[79] Corson M. A., Jones P. H., Davidson M. H. (2008). Review of the Evidence for the Clinical Utility of Lipoprotein-Associated Phospholipase A2 as a Cardiovascular Risk Marker. *American Journal of Cardiology*.

[80] Ali M., Madjid M. (2009). Lipoprotein-associated phospholipase A2: a cardiovascular risk predictor and a potential therapeutic target. *Future Cardiology*.

[81] Fèvre C., Bellenger S., Pierre A.-S. (2011). The metabolic cascade leading to eicosanoid precursors - Desaturases, elongases, and phospholipases A2 - Is altered in Zucker fatty rats. *Biochimica et Biophysica Acta (BBA) - Molecular and Cell Biology of Lipids*.

[82] Mai C., Wang B., Wen J., Lin X., Niu J. (2014). Lipoprotein-associated phospholipase A2 and AGEs are associated with cardiovascular risk factors in women with history of gestational diabetes mellitus. *Gynecological Endocrinology*.

[83] Insenser M., Montes-Nieto R., Angeles Martinez-Garcia M., Escobar-Morreale H. F. (2016). A nontargeted study of muscle proteome in severely obese women with androgen excess compared with severely obese men and nonhyperandrogenic women. *European Journal of Endocrinology*.

[84] Stuart C. A., Stone W. L., Howell M. E. (2016). Myosin content of individual human muscle fibers isolated by laser capture microdissection. *American Journal of Physiology-Cell Physiology*.

[85] Liu X., Takeda N., Dhalla N. S. (1997). Myosin light-chain phosphorylation in diabetic cardiomyopathy in rats. *Metabolism - Clinical and Experimental*.

